# Assessment and comparative analysis of a rapid diagnostic test (Tubex^®^) for the diagnosis of typhoid fever among hospitalized children in rural Tanzania

**DOI:** 10.1186/1471-2334-11-147

**Published:** 2011-05-24

**Authors:** Benedikt Ley, Kamala Thriemer, Shaali M Ame, George M Mtove, Lorenz von Seidlein, Ben Amos, Ilse CE Hendriksen, Abraham Mwambuli, Aikande Shoo, Deok R Kim, Leon R Ochiai, Michael Favorov, John D Clemens, Harald Wilfing, Jacqueline L Deen, Said M Ali

**Affiliations:** 1 Translational Research Division, International Vaccine Institute, Seoul, Korea; 2 Laboratory Division, Public Health Laboratory (Pemba) - Ivo de Carneri, Chake Chake, Tanzania; 3 Amani Centre, National Institute for Medical Research, Tanga, Tanzania; 4 Joint Malaria Program, Tanga, Tanzania; 5 Asia Pacific Malaria Elimination Network (APMEN), Menzies School of Health Research, Casuarina, Australia; 6 Teule Hospital, Muheza, Tanga, Tanzania; 7 Oxford Research Unit, Mahidol University, Bangkok, Thailand; 8 Biocenter, University of Vienna, Vienna, Austria

**Keywords:** Salmonella, Tubex^®^, Widal, Africa, Rapid Diagnostic Test

## Abstract

**Background:**

Typhoid fever remains a significant health problem in many developing countries. A rapid test with a performance comparable to that of blood culture would be highly useful. A rapid diagnostic test for typhoid fever, Tubex^®^, is commercially available that uses particle separation to detect immunoglobulin M directed towards *Salmonella *Typhi O9 lipopolysaccharide in sera.

**Methods:**

We assessed the sensitivity and specificity of the Tubex test among Tanzanian children hospitalized with febrile illness using blood culture as gold standard. Evaluation was done considering blood culture confirmed *S*. Typhi with non-typhi salmonella (NTS) and non - salmonella isolates as controls as well as with non-salmonella isolates only.

**Results:**

Of 139 samples tested with Tubex, 33 were positive for *S*. Typhi in blood culture, 49 were culture-confirmed NTS infections, and 57 were other non-salmonella infections. Thirteen hemolyzed samples were excluded. Using all non - *S*. Typhi isolates as controls, we showed a sensitivity of 79% and a specificity of 89%. When the analysis was repeated excluding NTS from the pool of controls we showed a sensitivity of 79% and a specificity of 97%. There was no significant difference in the test performance using the two different control groups (p > 0.05).

**Conclusion:**

This first evaluation of the Tubex test in an African setting showed a similar performance to those seen in some Asian settings. Comparison with the earlier results of a Widal test using the same samples showed no significant difference (p > 0.05) for any of the performance indicators, irrespective of the applied control group.

## Background

Typhoid fever remains a significant health problem in many developing countries. Estimates suggest an incidence rate of more than 21.5 million cases globally in the year 2000 [1]. Recent data from Tanzania mainland have found a strong variation of prevalence rates among blood culture positive isolates collected in local hospitals, ranging from 9% [2] to 21.4% [3] for *Salmonella *enterica serovar Typhi (*S*. Typhi), no data from Zanzibar are available to date. As the clinical picture of typhoid fever is often unspecific, misdiagnosis and insufficient or inadequate treatment are potential risks associated with the disease. In the absence of difficult-to-obtain bone marrow specimens, microbiologic culture of a blood sample is considered to be the current state-of-the art test for the diagnosis of typhoid fever even though its sensitivity may be as low as 40% [4,5]. Culture may take up to seven days and requires a well-run and equipped laboratory, which is often not available in settings with endemic typhoid fever. The widely in use Widal test provides a cost efficient alternative [6] for serological diagnosis, however its performance remains unsatisfying with sensitivity reported from Tanzania of 75% using blood culture as the gold standard and applying a cut off titer of 1:80 [7]. The test further requires the establishment of a local cut off titer prior to use which is complicated. Therefore, a rapid test with a performance comparable to that of blood culture would be desirable.

A rapid diagnostic test for typhoid fever, Tubex^® ^is commercially available that uses particle separation to detect immunoglobulin M (IgM) directed towards *Salmonella *enterica serovar Typhi (*S*. Typhi) O9 lipopolysaccharide in patient sera. Performance of the test has previously been evaluated in a number of studies in Asia but none in Africa. Using blood culture results for comparison, we assessed the sensitivity and specificity of the Tubex test among Tanzanian children hospitalized with febrile illness and compared our results with those from previous studies.

## Methods

For evaluation of the Tubex test, we used a selected subset of serum samples that was obtained for a fever surveillance study [2] from Teule Hospital in Muheza District, Tanzania. In order to accommodate the required sample size for the test validation, we included randomly selected and age-matched *Salmonella enterica *serotype Typhi (*S*. Typhi) positive serum samples from a second fever surveillance study conducted at Chake Chake Hospital in Pemba, Zanzibar. All samples were collected from children between the ages of 2 months to 14 years from 2008 to 2009.

At Teule Hospital in Muheza, sera and blood was collected for culture from children with a history of three days of fever, or a history of less than three days of fever but with at least one of the following severity criteria: respiratory distress; deep breathing; respiratory distress in combination with severe pallor; prostration; capillary refill ≥3 seconds; temperature gradient; systolic blood pressure <70 mm Hg; coma defined by Glasgow Coma Scale (GCS) ≤ 10 or Blantyre Coma Scale (BCS) ≤ 2; severe jaundice; history of two or more convulsions in the last 24 hours; blood glucose <3 mmol associated with clinical signs; neck stiffness; bulging fontanel; or oxygen saturation <90% [2].

At Chake Chake Hospital in Pemba, sera and blood was collected for culture from children with a recorded body temperature of >37.5°C for outpatients and any history of fever for inpatients. Duration of fever was not considered for study recruitment.

About 3 to 5 milliliters (ml) of blood (depending on body weight) was collected and inoculated in a BactALERT™ Pediatric-fan bottle (Teule Hospital) or a BacTec Peds PLUS ™/F bottle (Chake Chake Hospital) and incubated in the respective machine (BacT/ALERT 3D or BacTec 9050). Bacterial growth was evaluated following standard procedures.

The Tubex^® ^test (IDL - Sweden) was conducted according to the manufacturer's instructions, which are as follows. Forty-five microliters (μl) of antigen covered particles were added to the Tubex Reaction Well Strip and 45 μl of non-hemolyzed serum was added. After two minutes of incubation time, 90 μl of magnetic antibody coated solution was added, and the strip was sealed and shaken for two minutes. The strip was then placed on a magnetic tray for five minutes, separating the particles if a positive sample had been added. The resulting color change of the solution was read and categorized on a scale from 0 to 10. The results were interpreted as positive for scores of 4 or greater and as negative for scores of 2 or below as per the manufacturer's instructions. Samples with a color corresponding to the value of 3 were interpreted as indeterminate. All blood culture isolates from individuals that matched the inclusion criteria and that were not considered a contaminant were included in the analysis.

We performed the Tubex test on non-hemolyzed serum samples from the patients of the two surveillance studies who had blood culture-confirmed *S*. Typhi (defined as group 1), randomly selected cases of non-Typhi serotypes of *S. enterica *(NTS) (defined as group 2), and randomly selected cases with other (non-*Salmonellae*) pathogenic bacteria (defined as group 3). Staff members performing the Tubex test were blinded to the blood culture results.

For the analysis, sensitivity (true-positive rate) was defined as the probability that the Tubex test result will be positive when there is blood culture-confirmed typhoid fever (group 1) and specificity (true-negative rate) was defined as the probability that the Tubex^® ^test result will be negative when *S*. Typhi is not isolated from blood culture (groups 2 and 3). We conducted a secondary analysis using only group 3 as the control group. Comparison of test performance using different control groups was done using the Yates Chi-Square Test corrected for continuity.

We conducted a literature review in order to compare our findings with those from previous studies. We included studies of the Tubex test, which were identified by directly searching the MEDLINE database through PubMed. All articles since the first publication of the test [8] were included. We also conducted a supplementary search of references in retrieved articles. Abstracts were reviewed, and if relevant, the article was included.

A comparison of performance of the Tubex^® ^test with earlier published Widal test results obtained from the same samples was done using McNemar's Test for Correlated Proportions http://faculty.vassar.edu/lowry/VassarStats.html.

The fever surveillance studies at Chake-Chake and Teule Hospitals were approved by their respective local ethical review boards (Tanzania and Zanzibar), as well as by the International Vaccine Institute's Institutional Review Board. Written informed consent was obtained from legal guardians of all participants prior to any sample or data collection.

## Results

**Figure 1:**
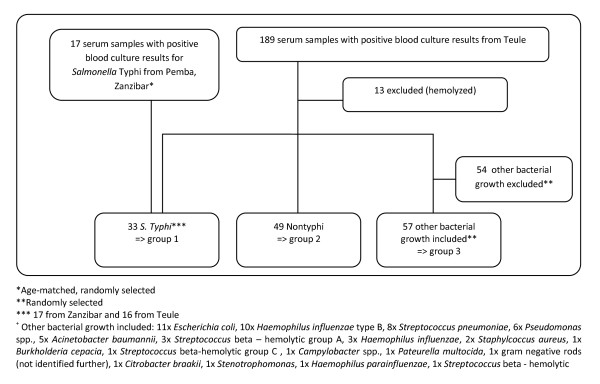
**Specimen assembly**.

A total of 139 samples were tested with Tubex. Thirty-three were found positive for *S*. Typhi in blood culture (group 1), 49 were culture-confirmed non-*S*. Typhi (NTS) infection (group 2), and 57 were other non-*Salmonella *infections that were not contaminants (group 3). Thirteen hemolyzed samples were excluded (Figure 1).

Of the 33 blood culture-positive *S*. Typhi cases, 26 had a positive Tubex result and were considered as true positives. Of the 106 blood culture confirmed NTS and non-salmonella cases (groups 2 and 3), 94 yielded a negative Tubex result and were considered as true negatives. Considering only the 57 non-*Salmonella *cases (group 3) as controls, resulted in 54 true negative cases.

Using groups 2 and 3 as controls showed a sensitivity of 79% and a specificity of 89% (Table 1). The same analysis was repeated excluding NTS from the pool of controls and showed 79% and 97% for sensitivity and specificity, respectively. There was no significant difference in the test performance using the two different control groups (all were p > 0.05 using the Chi square test).

**Table 1:** Performance of Tubex^® ^using group 1 as true positives and two different control groups as true negatives

	Control Group	
	Group 2 + 3*	Group 3*

**Sensitivity (95% CI)**	0.79 (0.52-0.81)	0.79 (0.62-0.90)
**(absolute numbers)**	(26/33)	(26/33)

**Specificity (95% CI)**	0.89 (0.81-0.94)	0.97 (0.85-0.99)
**(absolute numbers)**	(94/106)	(94/97)

*Group 1 = *S*. Typhi (n = 33), Group 2 = all non-yphi Salmonella (n = 49), Group 3 = all blood culture-positive non-*Salmonella *cases (n = 57)

A total of 14 articles were retrieved and evaluated for inclusion into the review. All of the reported studies were performed in Asia; none in Africa. A total of six articles were excluded: two evaluated the test for non-typhoidal *Salmonella *[9] or S. Paratyphi [10], three did not evaluate the sensitivity and specificity of the test [11-13], and one was a letter to the editor [14]. Thus, eight publications were included in the review (Table 2). Five of the included articles reported findings of test performance that were similar to our results [15-19]. Two publications showed considerably lower sensitivity and specificity [20,6], and one reported higher values [8] (Table 2).

**Table 2:** Comparison of the performance of the Tubex^® ^test from published reports

**Author**	**Year**	**Journal**	**Sample Size**	**Location**	**Tubex^® ^cut off **	**Sens**	**Spec**	**True neg**. **definition**	**Reader**	**Gold standard**	**Study population**
Ley, B. et al	This paper	This Journal	139	Tanzania	>4	79%	89%	All non-typhi bacteriamia	Investigator	Blood Culture (BACTEC)	>2 months + >37.5° (inpatients) & history of fever (outpatients)
									
			88			79%	97%	All non-salmonella bacteriamia			

Naheed, A. et al	2008	Diagn Microbiol Infect Dis.	867	Bangladesh	≥4	60%	58%	Other confirmed bacteremia	ICDDRB lab	Manual Blood Culture	Active surveillance Temp ≥38°C
									
						60%	64%	Blood culture neg & other bacteremia			

Rahman, M. et al	2007	Diagn Microbiol Infect Dis.	243	Bangladesh	>4	91.2%	82.3%	Other febrile patients	ICDDRB lab, min. 2 independent lab techs	Manual Blood Culture	Outpatients, all ages with history of fever
									
						No. Pos	89.5%	Healthy subjects			Healthy subjects

Dong, B. et al	2007	Epidemiol. Infect.	1732	China	≥2	100%	43%	Paratyphoid cases	-	Blood culture (BACTEC)	Age 5-60 with reported history of fever for 3 days
									
					≥4	69%	95%				
									
					≥6	62%	95%				
									
					≥8	23%	100%				
									
					≥10	15%	100%				

Kawano, R. L. et al	2006	JCM.	177	Philippines	≥2	94.7%	80.4%	Blood culture neg.	n/A	Manual Blood Culture &BACTEC	Clinically suspected typhoid cases

Dutta, S. et al	2006	Diagn Microbiol Infect Dis.	495	India	≥4	56%	88%	Paratyphoid and malaria cases	n/A	Blood Culture BACTEC	Outpatients, all ages, Pat with history of fever for 3 days

Ohlsen, S. J. et al	2004	JCM.	79	Vietnam	According to protocol	78%	94%	Other lab-confirmed febrile illnesses	n/A	Manual Blood Culture/BACTEC	Pat ≥3 year and history of ≥4 day fever

House, D. et al	2001	JCM.	127	Vietnam	>2	87%	76%	Febrile hospitalized patients	labtech	Culture	Children and adults

Lim et al	1998	JCM.	105	Hong Kong & Malaysia	>2	100%	100%	Healthy individuals and pat with other bacterial diseases and autoimmune disease	labtechs	Culture confirmed (56% of pos.), clinical picture, various other tests	Clinical picture, culture confirmed,

## Discussion

We found Tubex has a sensitivity of 79% using either control group (95%CI: 52-81% for groups 2 and 3, and 62-90% for group 3 only) and a specificity of 89-97% (95%CI: 81-94% for groups 2 and 3 and 85-99% for group 3 only) irrespective of control group. To our knowledge, this is the first evaluation of the test in an African population. Our results were similar to those observed in previous studies (five out of eight studies) in Asia assessing the performance of the test [15-19], though Kawano *et al. *[17] and House *et al. *[19] used a lower cut-off titer than is recommended in the manual. In contrast, two studies [20,6] found the performance of Tubex to be poorer than our findings, despite using a similar cut-off value, gold standard, and inclusion criteria. The extremely good performance of Tubex observed by Lim *et al. *[8] has not been reproduced since.

An important limitation of this study is that the sera are combined from two different patient populations and the purposeful selection of samples included in the three groups. During the preparation of the study, we calculated the sample sizes of true positive sera and true negative sera that are required for validation of the Tubex test and for comparison with the Widal test performance. The number of true positive sera from either hospital alone was insufficient for the validation. Thus, we included *S*. Typhi blood culture-confirmed sera from Pemba. Analysis of the results by hospital was not possible because of insufficient sample size.

In a sub-analysis in assessing cross reactivity with NTS, blood culture-confirmed NTS cases were considered as true positives, and all other positive isolates, excluding *S*. Typhi, were considered as true negatives. In this sub-analysis Tubex had a sensitivity of 18% and a specificity of 95% (analysis not shown).

Comparison with a Widal test that was earlier conducted using the same samples [7] revealed no significant difference (p > 0.05) for any of the performance indicators, irrespective of the applied control group. But compared to the Widal test, Tubex is easier and quicker to perform. The Widal test requires 16 - 20 hours until the results are obtained while the complete procedure for the Tubex test is approximately 20 minutes. Tubex is more expensive at approximately 2.15 USD per test compared to <0.80 USD per test for the Widal tube agglutination test [6].

Interpreting the Tubex test results was found to be difficult and the results were prone to inter - reader variation. Assessing the color change according to the provided color scale requires experience and standardized good lighting conditions. The Tubex test can only be applied to non-hemolyzed and non-icteric serum samples, thus limiting its general application. However Tam *et al. *[12] have described a method that includes a washing step and thereby addresses the problem of turbid serum. This method requires double the amount of antigen-coated particles as well as glycine buffered saline (GBS), thereby increasing the price per sample to approximately 4.50 USD and reducing its feasibility as an easy-to-perform test. While neither of the tests can be performed by untrained staff, interpretation of results is considered easier for the Widal test compared to Tubex.

## Conclusion

The advantages of Tubex over the Widal test and the gold standard of blood culture is the short time it requires to obtain a result, and it does not require establishing a local cut-off value as with the Widal. In settings that can afford the relatively high cost of Tubex and that require instant individual diagnoses to support the clinical diagnosis of typhoid fever, Tubex is superior to the Widal tube agglutination test. For screening and surveillance purposes, as well as in settings with limited financial and technical resources, the Widal tube agglutination test is a possible alternative that can provide a similar performance as Tubex at a lower cost though it requires more time. Our evaluation of Tubex showed that any result must be handled with precaution. Results should be considered as indicative, not confirmatory. The test may be used to exclude disease though. In conclusion, the need for a reliable, fast, cheap, and easy-to-apply rapid diagnostic test for typhoid fever remains in high demand.

## Competing interests

The authors declare that they have no competing interests.

## Authors' contributions

BL performed the TUBEX test, analyzed results and wrote the manuscript, KT performed TUBEX tests, literature search and contributed to the manuscript, SMA supervised the laboratory work in Pemba, GM was in charge of the implementation and study management in Teule, LvS provided scientific support to study staff and contributed to the manuscript, BA supervised the laboratory work in Teule, ICEH was involved in the clinical care of patients, AM was in charge of data management, AS performed blood culture procedures, DRK performed statistical analyses, RLO provided scientific support to the manuscript, MF provided scientific support to the manuscript, JDC provided scientific support to the manuscript, HW provided scientific support to the manuscript, JLD provided major scientific support to the manuscript and was involved in the clinical care of patients, SMA provided scientific support to the manuscript and the study in Pemba. All authors have read and approved the final manuscript.

## Pre-publication history

The pre-publication history for this paper can be accessed here:

http://www.biomedcentral.com/1471-2334/11/147/prepub
